# Innovative wood use can enable carbon-beneficial forest management in California

**DOI:** 10.1073/pnas.2019073118

**Published:** 2021-11-22

**Authors:** Bodie Cabiyo, Jeremy S. Fried, Brandon M. Collins, William Stewart, Jun Wong, Daniel L. Sanchez

**Affiliations:** ^a^Energy and Resources Group, University of California, Berkeley, CA 94720;; ^b^Pacific Northwest Research Station, US Department of Agriculture Forest Service, Portland, OR 97205;; ^c^Center for Fire Research and Outreach, University of California, Berkeley, CA 94720;; ^d^Pacific Southwest Research Station, US Department of Agriculture Forest Service, Davis, CA 95618;; ^e^Department of Environmental Science, Policy, and Management, University of California, Berkeley, CA 94720

**Keywords:** forests, wildfire mitigation, harvested wood products, carbon balance

## Abstract

Responsible stewardship of temperate forests can address key challenges posed by climate change through sequestering carbon, producing low-carbon products, and mitigating climate risks. Forest thinning and fuel reduction can mitigate climate-related risks like catastrophic wildfire. These treatments are often cost prohibitive, though, in part because of low demand for low-value wood “residues.” Where treatment occurs, this low-value wood is often burned or left to decay, releasing carbon. In this study, we demonstrate that innovative use of low-value wood, with improved potential revenues and carbon benefits, can support economical, carbon-beneficial forest management outcomes in California. With increased demand for wood residues, forest health–oriented thinning could produce up to 7.3 million (M) oven-dry tonnes of forest residues per year, an eightfold increase over current levels. Increased management and wood use could yield net climate benefits between 6.4 and 16.9 million tonnes of carbon dioxide equivalent (M tCO_2_*e*) per year when considering impacts from management, wildfire, carbon storage in products, and displacement of fossil carbon-intensive alternatives over a 40-y period. We find that products with durable carbon storage confer the greatest benefits, as well as products that reduce emissions in hard-to-decarbonize sectors like industrial heat. Concurrently, treatment could reduce wildfire hazard on 4.9 M ha (12.1 M ac), a quarter of which could experience stand-replacing effects without treatment. Our results suggest that innovative wood use can support widespread fire hazard mitigation and reduce net CO_2_ emissions in California.

## Significance

Natural carbon sinks can help mitigate climate change, but climate risks—like increased wildfire—threaten forests’ capacity to store carbon. California has recently set ambitious forest management goals to reduce these risks. However, management can incur carbon losses because wood residues are often burnt or left to decay. This study applies a systems approach to assess climate change mitigation potential and wildfire outcomes across forest management scenarios and several wood products. We find that innovative use of wood residues supports extensive wildfire hazard reduction and maximizes carbon benefits. Long-lived products that displace carbon-intensive alternatives have the greatest benefits, including wood building products. Our results suggest a low-cost pathway to reduce carbon emissions and support climate adaptation in temperate forests.

Climate change poses substantial challenges to managing temperate forests, particularly in California (1, 2). Due to extensive timber harvesting and fire exclusion in the 20th century, California forests are younger, denser, and more homogeneous than historical conditions (3, 4). These changes have left California forests vulnerable to large-scale disturbances like drought, insects, disease, and wildfire. As in other temperate forests, California forests are at risk from increasing fire severity and frequency driven by climate change (5–7). Extreme wildfire events with large proportions of stand-replacing effects have become more common and pose an existential threat to forest ecosystems and their capacity to sequester carbon, particularly on federal lands (1, 8–13).

At the same time, recent work has emphasized the potential of forests to help meet climate goals in the near and long term (14–17). Still, tremendous uncertainties exist around aligning forest treatment and climate goals. Estimates of how forest treatment will impact net carbon emissions from temperate forests vary substantially (10, 18, 19). There is broad consensus that more efficient use of harvested wood can improve the carbon balance of management, but different wood products vary substantially depending on production emissions, substitution benefits, and end-of-life emissions (20–23).

In response to increasing wildfire risk, California’s Forest Climate Action Team and the State of California have set a goal to reduce wildfire hazard on 1 million (M) acres (0.4 M ha) of public and private forest per year (24). These plans invoke fuel reduction treatments, timber harvest, and expanded use of harvested wood products. Active management—like prescribed fire and mechanical thinning—can mitigate wildfire impacts and provide many co-benefits (25, 26). However, these treatments are often costly even where the sale of larger harvested trees (sawtimber) is possible. Furthermore, fuel treatment effectiveness depends primarily on the removal of small trees, which comprise most of the “ladder” fuels in forests (27). Sale of small trees and residues (e.g., as biomass chips or pulpwood logs) could offset some treatment costs, but present market demand is limited. As a result, large amounts of low-value wood are left to decay or are burned after treatment, releasing stored carbon to the atmosphere. We propose that an alternative fate for this wood may enable expanded treatment and the flexibility to manage for multiple goals.

**Fig. 1.**
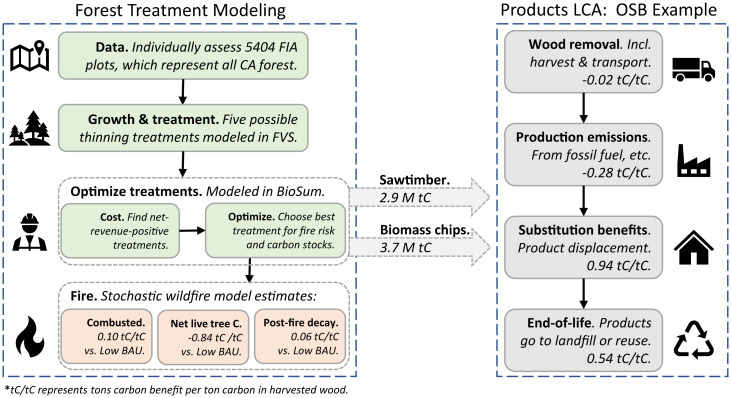
Modeling framework, system boundaries, and example results for one product, oriented strand board (OSB). Product carbon benefits (*Right*) are specific to OSB, while in-forest carbon fluxes (*Left*) are common to all products in the IWP scenario. Carbon benefit values presented are cumulative over 40 y. Refer to *SI Appendix*, *SI Methods* for a complete description of all steps above.

**Fig. 2.**
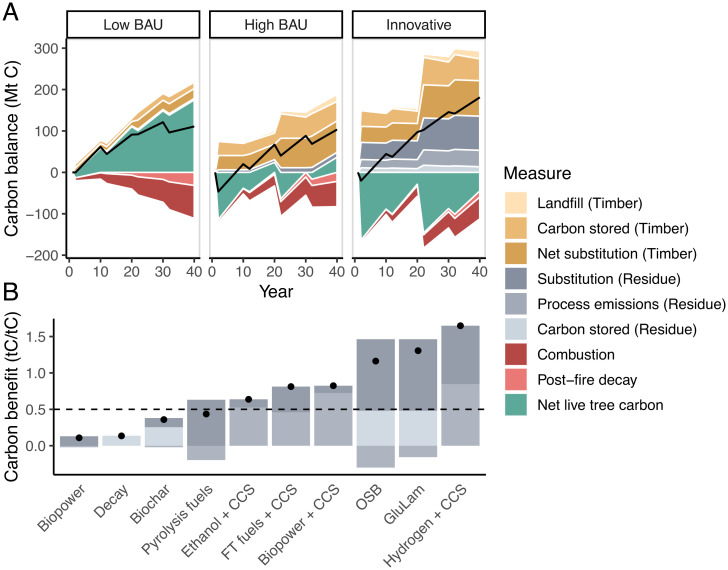
Life cycle, forest carbon balance across (*A*) three scenarios and (*B*) several technology pathways. Net carbon values are represented by dots in *B* and black lines in *A*. In *B*, the dotted line represents the threshold used to select the suite of technologies in IWP. Net live-tree carbon values are relative to carbon stocks in year zero, and large decreases are associated with harvest events. In Low BAU, we model management only on corporate land where potentially profitable (net revenue >$2,500/ha). In High BAU, we model management wherever it is net revenue positive with a delivered residue price of $0. In Innovative (IWP), we model management wherever it is net revenue positive with a delivered residue price of up to $100/ODT. The treatment area under IWP defines the study area for High and Low BAU, which include untreated forest.

In this study, we investigate how a robust market for forest residues could affect the scale and impact of forest treatment in California. First, we model forest health–oriented thinning treatments (*Materials and Methods*) and potential wildfire outcomes on California’s public and private timberland with Forest Inventory and Analysis (FIA) data (Fig. 1). We consider three management scenarios: 1) Business as Usual with Limited Management (Low BAU), 2) Business as Usual with Expanded Management (High BAU), and 3) Innovative Wood Products (IWP). In the IWP scenario, the potential revenue generated from IWP supports increased management over either BAU. Second, we examine the carbon benefits of several pathways for harvested wood using attributional lifecycle assessment, including production emissions, carbon storage, substitution of carbon-intensive alternatives, and end-of-life emissions. In Fig. 2*A* , we present the net carbon balance from expanded forest management and wood product markets in California.

## Results

### Baseline Scenarios.

The Low BAU scenario represents a low-management future similar to, but not the same as, current practice in California (see *Discussion*). In Low BAU, we assume no thinning in both public and private (i.e., family owned) forests. On corporate-owned land, we model thinning on the 0.8 M ha (2 M ac) where net revenue is >$2,500/ha without revenue from forest residues. Under this management scenario, 1.6 M oven-dry tonnes (ODT) (4.1 M m^3^) per year of sawtimber are harvested over the next 40 y on average. For comparison, California produced 3.8 M m^3^ of sawtimber per year on average over the past decade (13). The Low BAU scenario is characterized by both high fire hazard and high rates of carbon storage in untreated forest. Accounting for wildfire occurrence and effects via stochastic simulation, this forest land will sequester 0.89 ± 0.02 tC·ha^−1^ · y−1 over the next 40 y (± indicates 95% CI from a Monte Carlo simulation). This value is close to previous estimates for temperate coniferous forests in the western United States (e.g., ref. 28). Direct emissions from fire are 0.40 ± 0.01 tC · ha^−1^ · y^−1^, but postfire decay increases total emissions by 0.17 ± 0.007 tC · ha^−1^ · y^−1^ over 40 y (*SI Appendix*, Fig. S4). We present alternative formulations of BAU in the SI, with similar results.

**Fig. 3.**
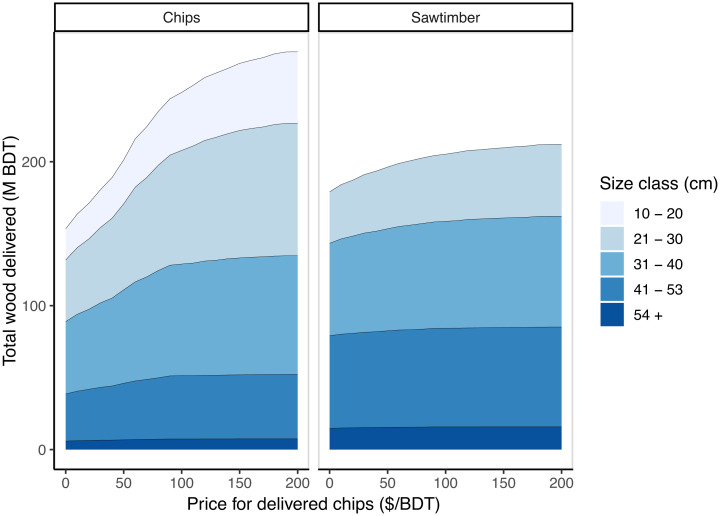
Wood availability at increasing delivered forest residue prices by DBH class. Residues include small trees, tops and branches from larger trees harvested for sawtimber, and entire trees of noncommercial species.

In the High BAU scenario, we consider the impact of maximizing the scale of management without subsidy (i.e., where net revenue is positive) and without revenue from forest residues. In this scenario, it is possible to manage 3.3 M unique ha over 40 y (8.1 M ac) on both public and private land. The resultant flow of harvested sawtimber is nearly three times larger than in Low BAU at 5.12 M ODT (13 M m^3^) per year, comparable to historical production volumes (13). Most of this wood comes from trees smaller than 53 cm diameter at breast height (DBH) (Fig. 3). In addition, 4.4 M ODT of forest residues are technically available in this scenario. Without a price on forest residues sufficient to recoup removal and transport, however, it is likely that this wood would be left to decay or burned in-forest. Where subsidies exist, forest residues may be sent to biopower facilities. Compared to Low BAU, increased management leads to a reduction in wildfire-related emissions: direct emissions from wildfire are 0.32 ± 0.01 tC · ha^−1^ · y^−1^, and decay adds 0.12 ± 0.005 tC · ha^−1^ · y^−1^ over 40 y. While the High BAU scenario reduces wildfire hazard on more hectares than Low BAU, it poses two key challenges: 1) management of stands dominated by small trees can be cost prohibitive without subsidy, and 2) the combustion or decay of low-value wood conflicts with climate goals.

### IWP Scenario.

In the “IWP” scenario, we examine how innovative uses of forest residues can enable better economic and carbon outcomes from management. We assess several products that are commercially and technically mature and have an estimated market size equivalent to >1 M ODT wood per year in California (29). We estimate that low-carbon fuel and oriented strand board (OSB) production can justify delivered forest residue prices in excess of $100/ODT delivered (*SI Appendix*, Fig. S6), similar to other techno-economic analyses (30–32). In IWP, we assume a delivered price of up to $100/ODT, which supports management beyond what is economically possible in High BAU. Most forest residues are available at lower prices, however (Fig. 3).

With this additional revenue, 4.9 M ha (12.1 M ac) of forest can be managed over the next 40 y without subsidy. Some of this area is treated more than once, so on average, ∼0.2M ha (∼0.5M ac) of forest can be treated each year. Most of this treatment is technically possible in the first two decades (*SI Appendix*, Fig. S1). We estimate that at this price, thinning could produce 7.3 M ODT of forest residues and 14.8 M m^3^ (5.7 M ODT) of sawtimber annually over 40 y. This would represent a nearly eightfold increase over current forest residue supply and a fourfold increase in sawtimber production (13, 33). Increased residue prices do not appreciably increase sawtimber harvest: while residue availability increases sharply by 62% with prices up to $100/ODT, sawtimber availability only increases in the smallest merchantable diameter classes (Fig. 3). Even a residue price of $200/ODT would only increase sawtimber availability by 18% compared to no residue price. At $100/ODT, a relatively small fraction (40 million ODT, 19%) of forest residues comes from small trees (10 to 20 cm DBH). Most residue is a byproduct of whole-tree harvest of larger trees and the entirety of trees of noncommercial species.

**Fig. 4.**
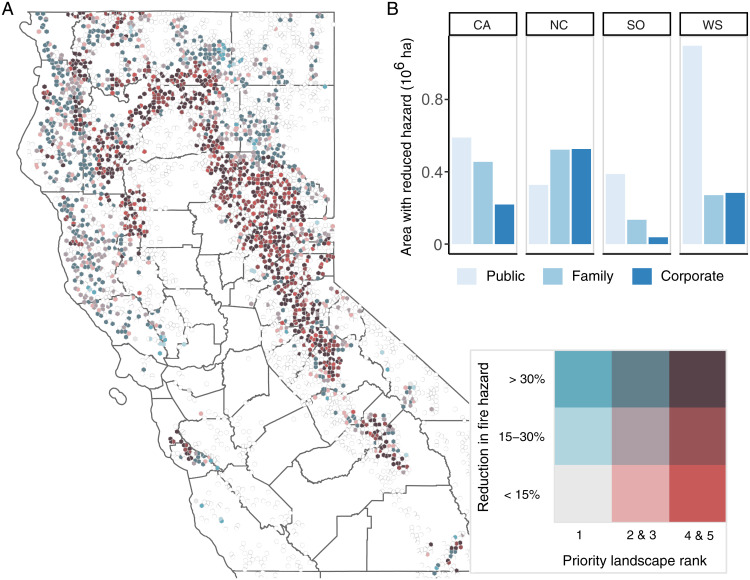
Fire hazard reduction in the IWP scenario in (*A*) CalFire Fire Priority Zones and (*B*) summed across the study area. Reduction in fire hazard is defined as the difference in basal area mortality fraction with and without treatment in the event of a wildfire with severe fire weather. In *B*, each hexagon represents a single FIA plot, which is statistically representative of a larger area of forest (usually ∼2,000 to 2,500 ha). Empty hexagons represent untreated plots, and county boundaries are shown in the background. In *B*, values are grouped by FVS Variants, where CA is Central California, NC is North Coast, SO is Northeast California, and WS is Western Sierra. Colors represent ownership groups, in which “Family” is noncorporate private land.

In IWP, it’s possible to treat 1.3 M ha (3.1 M ac) that could experience stand-replacing wildfire effects (>95% mortality) without treatment, reducing potential basal area mortality by 28 ± 1% on average in those stands (*SI Appendix*, Fig. S3). Of all the area treated, 47% occurs on landscapes designated by CalFire as a high-priority (Zones 4 and 5) for reducing wildfire risk to ecosystem services (Fig. 4). Mean annual combustion emissions from wildfire are 0.27 ± 0.01 tC · ha^−1^ · y^−1^, and postfire decay adds 0.07 ± 0.005 tC · ha^−1^ · y^−1^. This represents a reduction in fire-driven emissions of 39% over Low BAU and 19% over High BAU.

### Wood Product Life Cycle Analysis.

For the current mix of California sawtimber end uses (13), we estimate a net substitution factor of 0.75 tC benefit per tC harvested (tC/tC), in which “net” is the sum of production emissions and substitution of carbon-intensive alternatives. This value is slightly higher than estimated for Canada (34), because a larger fraction of timber products in California are used in buildings. It is lower than in similar studies, though, partially because building operational emissions are excluded (34). Wood that displaces steel and concrete has the largest carbon benefits of any use studied here. For this reason, we consider the effect of diverting all additional (versus Low BAU) sawtimber produced in IWP to multifamily and multiuse buildings. This “IWP+Housing” scenario represents a future in which affordable, medium-density housing is prioritized. In IWP+Housing, the net substitution factor is 1.75 tC/tC because of increased steel and concrete substitution. This value is similar to net substitution factors found for other regions (34, 35), despite our optimistic wood-use assumptions. When we include the end-of-life (modeled to 40 y), we find a weighted net carbon benefit of 1.35 tC/tC for all sawtimber products. In the IWP+Housing scenario, it is 2.35 tC/tC.

For forest residue products, carbon benefits vary substantially (Fig. 2*B*). Biopower, currently the most common use of forest residues in California, has a low carbon benefit (0.11 tC/tC) relative to more innovative technologies, primarily because of the absence of CO_2_ storage and the displacement of relatively clean California grid electricity. Conversely, technologies with a large fraction of carbon storage have the greatest benefits. Biopower with carbon capture and storage (CCS) has a comparatively high carbon benefit (0.81 tC/tC), because a large portion of the emitted CO_2_ is captured and stored. Hydrogen with CCS, glue-laminated timber (GluLam), and OSB have the highest carbon benefits (1.18 to 1.65 tC/tC) of any of the studied products because of both high substitution benefits and carbon storage in wood products or via CCS. Further, these three products would all reduce emissions in “hard-to-abate” sectors like cement and industrial heat. While all these residue-based products are technically feasible, they rely on different forest residue components. OSB and GluLam, for example, require small-diameter (pulpwood) logs while hydrogen production can use mixed biomass that includes leaves and bark. In IWP, we present an equal mix of only the products that both exceed a 0.5 tC/tC carbon benefit threshold and could use mixed biomass (fuels) or pulpwood logs (OSB and GluLam) at commercial scale (Fig. 2*B*).

### Net Climate Impacts.

We estimate the economy-wide net climate impact of management by combining in-forest carbon changes with harvested carbon benefits (Fig. 2*A*). Thus, the net carbon balance is a combination of sequestration, storage, emissions, and avoided emissions. In all three scenarios, the forest sector is a net carbon sink. In Low BAU and High BAU, we find similar net carbon benefits of 10.2 M and 9.5 M tCO_2_*e* per year, respectively, over 40 y. The IWP scenario has a larger carbon benefit of 16.6 M tCO_2_*e* per year. In all three scenarios, traditional sawtimber products play an important role in supporting a positive net carbon balance of management. The IWP scenario, though, suggests clear benefits from innovative use of forest residues and sawtimber. In terms of climate goals, shifting from Low BAU to IWP confers a net climate benefit of 6.4 M tCO_2_*e* per year, primarily because of innovative forest residue use. IWP+Housing yields a higher net benefit of 27.1 M tCO_2_*e* per year, or 16.9 M tCO_2_*e* per year over Low BAU, largely due to substitution of steel and concrete with sawtimber. On a timescale relevant to California’s immediate climate goals (2045), the IWP+Housing Scenario has the most pronounced, immediate benefits (*SI Appendix*, Fig. S5). In sum, innovative wood use may be critical to achieving California’s dual goals of reducing both wildfire hazard and CO_2_ emissions.

## Discussion

Our results suggest that efficient wood use can play an important role in establishing California’s forests as a resilient, long-term carbon sink. We find that IWP would increase the scale of management and carbon benefits from forest residues that would otherwise decay or be burned. These products can simultaneously advance existing forest management and climate goals in California. Below, we review our results in the context of forest management, innovative wood-use technologies, and climate policy. We also highlight that, although this study integrates several critical elements of a complex system, there are important limitations. This analytical framework might serve as a template and a starting point to further investigate the complex interface between wood use and management in high-disturbance forests. Further, large-scale forest treatments like those discussed here may have unforeseen consequences. Investigating ecological outcomes not analyzed in this study, such as the comparative impacts of wildfire and expanded forest treatments on ecosystem services like biodiversity, would be a fruitful area of inquiry to extend this framework.

In this study, we emphasize thinning and surface fuel treatments aligned with California guidelines (*Materials and Methods*). These treatments promote multiple ecosystem benefits and a return to historical forest structure by reducing stand density and retaining the largest, most fire-resistant trees (3, 4, 36). Forest management plans are necessarily context dependent and will depart, to varying extents, from those we assumed here. It’s also likely that future management plans will require novel approaches to respond effectively to climate conditions without historical precedent (37, 38). Management planning may best be conceived as a proactive, adaptable process in order to meet multiple social and ecological goals under changing environmental conditions (37, 38). However, we find that across most timberland in California, carbon-beneficial treatment is not feasible without including wood products. Innovative use of wood may be necessary to ensure that wildfire-motivated treatments yield climate benefits. This strategy complements others that emphasize reforestation or prolonged retention of larger trees to aid climate goals (14, 15, 17).

Innovative wood use has two primary value propositions in California: increasing revenues from harvested wood and improving the carbon balance of forest management. Two promising classes of products have emerged in recent reviews: low-carbon and carbon-negative fuels and engineered structural wood products (e.g., mass timber) (29, 32, 39). Low-carbon fuels derived from woody biomass show economic promise because of supportive state and federal fuel policy, including the California Low-Carbon Fuel Standard. Multiple large-scale transportation fuel projects using California wood and biomass but located in neighboring states plan to commission plants in 2022. If additional facilities are instead sited in California, low-carbon and carbon-negative fuels can drive additional regional economic development benefits.

Mass timber products like cross-laminated timber (CLT) and GluLam are uncommon in the United States but have been widely adopted in European markets. Other engineered wood products like OSB, which can be made from pulpwood logs, are widely used but not produced in the western United States (40). Specific engineered wood products may have relatively higher substitution benefits (e.g., I-beams produced with OSB) or higher carbon storage density (e.g., CLT). These products often displace steel and concrete and would support our IWP+Housing scenario, which has the greatest net carbon benefit of any scenario. The recent inclusion of CLT in California’s building code may encourage widespread adoption and production. However, further research should verify the suitability of small-diameter wood, low-quality wood, and California tree species as feedstocks for these products.

In these cases, climate policies can play a critical role. California’s Low-Carbon Fuel Standard, for example, provided a financial incentive between $160 and 192/tCO_2_ abated in 2018 through 2019 and was recently extended through 2030. Revenue from carbon payment programs like this enable the financial viability of innovative wood use. Similarly, the state has recently adopted other performance-based climate policies, such as Buy Clean California, that could drive use of wood building products. Investment mechanisms, like the new Climate Catalyst Fund, can also play an important role in defraying upfront costs, although these funds will need to grow to support facilities with higher capital costs (e.g., OSB). Those facilities may also require long-term supply contracts to ensure that capital costs will be recovered. Finally, workforce development initiatives could support the rapid scaling of forest treatments. Such policies may help achieve the central goal of the state’s Forest Carbon Plan: to firmly establish California’s forests as a more resilient and reliable long-term carbon sink (24).

### Study Limitations.

In our scenario analysis, we suggest Low BAU and High BAU as baseline scenarios. While neither of these scenarios are a perfect representation of reality, we expect that they bracket a range of likely futures without the influence of innovative wood use. We use Low BAU as the basis for comparison because it most closely approximates the current state of forest management in California, with high rates of active management under corporate ownership and much less on public- and family-owned forests. We also consider an alternative BAU that includes a more representative mix of public and private management but does not materially change the results presented here (*SI Appendix*, *SI Methods and Results*). Alternatively, increased interest in wildfire hazard reduction and related policy changes may yield a future more similar to High BAU.

In this study, we have employed an attributional life cycle analysis (LCA) approach, which includes the physical flows to and from a given system. However, it is unlikely that wood harvested in California will be exclusively used in California, and the consequences of an influx of wood products into the global market may have unforeseen outcomes. Localized policy, like California’s Green Procurement Strategy, or policy that supports substituting wood products for carbon-intensive alternatives, may promote greater carbon benefits from wood harvested in the state without displacing wood products elsewhere. Although the LCA values used here represent current technology, the carbon benefits of these products may increase or decrease over the modeling period. Substitution benefits may change significantly as the mix and carbon intensity of displaced products evolves.

Predictions of future wildfire occurrence and outcomes are inherently uncertain (41). In our simulations, growth and wildfire emissions vary substantially depending on which forest plots burn and when they burn. This effect is most pronounced in Low BAU, in which large amounts of carbon are stored in untreated forest, but the stability of that carbon is highly uncertain. The values that parameterize decay and combustion have a large effect on wildfire emissions as well. The parameters we use exclude non-CO_2_ climate forcers and may underestimate actual wildfire emissions, limiting the carbon benefits of treatment. Further, we model forest growth in the Forest Vegetation Simulator (FVS), which is known to underestimate mortality and thus overestimate growth. We do not model the impact of non-fire climate effects like increased incidence of drought, insects, disease, or CO_2_ fertilization. Nor do we consider persistent shifts in vegetation (e.g., from timberland to shrubland). In aggregate, we likely overestimate forest carbon stability and underestimate the carbon benefits of forest treatment (1).

## Materials and Methods

A full documentation of the methods used to produce this work can be found in *SI Appendix*, *SI Methods and Results*. Here, we present a brief summary of those methods.

### Management.

This analysis applies the FIA BioSum modeling framework (42) to understand management outcomes on California timberland. We rely on data collected from 5,404 field-sampled FIA plots between 2005 and 2016 that represent ∼13.4 M ha (33 M ac) of California forest land. We refine this forest land sample to limit our analysis to forests that are classified as timberland and as one of four common California forest types: mixed conifer, Douglas fir, true fir, and ponderosa pine. We consider the three ownership types that account for nearly all of California’s timberland: corporate, noncorporate private (“family”), and National Forest System (“public”). We exclude land federally reserved from management and land administered by state and local government.

We model forest growth, management, and potential fire outcomes over 40 y with FVS and the associated Fire and Fuels Extension (FFE). For each FIA plot, we simulate five forest treatments (*SI Appendix*, Table S1) designed to represent forest restoration–motivated management compatible with the provisions of the Sierra Nevada Forest Plan. The treatments differ with respect to thinning style (from below or across diameter classes), maximum size of trees allowed to be harvested, and treatment of surface fuels. Each treatment reduces basal area by a maximum of 33%. Treatments implement thinning with a whole-tree harvest system using either a mechanical harvester (for DBH < 53 cm) or manual felling. After thinning, surface fuels are treated with either prescribed fire or lop and scatter. We also simulate a “Grow Only” alternative to represent untreated forest. Subsequently, we evaluate costs and revenues for each treatment using BioSum. Sawtimber values are based on California Board of Equalization rates, and in the IWP scenario, residues have a maximum delivered value of $100/ODT, although most can be delivered at lower prices (*SI Appendix*, *SI Methods and Results*). Residues include small trees (DBH < 20 cm), tops of larger trees, branches, and the entirety of noncommercial species of all sizes. We conduct a multicriteria optimization in BioSum to choose a treatment that is net revenue positive, reduces fire hazard, and maximizes live-tree carbon at the end of 40 y. Based on this optimization, BioSum calculates quantities of sawtimber and forest residues that could be delivered to an existing network of processing facilities.

### Wildfire Modeling.

To understand the in-forest carbon balance of management, we stochastically simulate wildfire based on static potential fire outcomes predicted by FVS-FFE. These potential fire outcomes are modeled for each year independently and represent “what-if” fire hazard metrics. We develop a stochastic model to understand how these potential outcomes would manifest under a fire regime consistent with contemporary and probable future fire activity. We run 5,000 Monte Carlo simulations for each management scenario to reflect the inherent spatial and temporal variability of wildfire. In each simulation, we randomize 1) how many plots burn, 2) which plots burn, and 3) when they burn. We assume a mean annual fire probability of 0.092%, which is slightly higher than historical conditions due to climate change (7, 12). We simulate both 90th and 97.5th percentile fire weather conditions, which are expected to increase in frequency during our modeling period (6, 43). These percentiles are associated with large wildfire occurrence in California forests, which account for an overwhelming majority of total burned area over recent decades (44). In this study, we present a mean of these two fire weather conditions. For each stand and simulated wildfire, we estimate sequestration and emissions associated with growth, fire, and decay. We predict postfire growth with a scalar function derived from predicted wildfire mortality by basal area and FVS growth projections. We parameterize both direct combustion and postfire decay with published values (45, 46). These parameters likely understate wildfire-induced emissions and, by extension, the carbon benefits of management (*SI Appendix*, *SI Methods and Results*).

### Life Cycle Analysis of Wood Products.

We rely on published values to model the cradle-to-grave carbon benefits for harvested wood across four categories: harvest and transport emissions, production emissions, substitution of carbon-intensive products, and product end-of-life (Fig. 1). We consider 1 tonne of harvested carbon as the primary unit of analysis. For forest residue-based products, we use data from LCA studies with feedstocks and system boundaries similar to what we model here. We only consider products that could use a portion or all of the feedstock modeled here (e.g., topwood, but not mixed biomass, for OSB; *SI Appendix*, *SI Methods and Results*). Where possible, we rely on data from studies using the Greenhouse Gases, Regulated Emissions, and Energy Use in Transportation (GREET) model (47). We normalize harvest and transport emissions across all products to be consistent with values used in GREET. Across all product pathways, we assume an average California grid carbon intensity of 225 g CO_2_e/kWh (48). The methods and assumptions for each product pathway are described in *SI Appendix*, *SI Methods and Results*.

For sawtimber, we adapt the methodology used by ref. 34 to the California market context. This approach yields an economy-wide displacement factor for sawtimber products including emissions from extraction, transportation, and production of a representative suite of building materials. In this study, we retain all values in ref. 34 except for product end uses, which are economy specific. We use historical California-specific end use data instead (13). In the IWP+Housing Scenario, 100% of increased sawtimber supply (over Low BAU) is assumed to be used in multiunit buildings, resulting in a larger net substitution factor (*SI Appendix*, Table S4). We conservatively assume that 24% of sawtimber is used for biopower and 75% is used in durable wood products, despite more carbon-beneficial uses for sawmill residues (49). We calculate a category-weighted mean half-life for all primary wood products of 38 y (*SI Appendix*, Table S4) (50). After primary use, we assume that 65% of retired wood products are sent to landfills, 25% to biopower facilities, and 10% are not collected (49). In the landfill, 90% of wood carbon is assumed to be permanently inert (51, 52), although this assumption has a limited effect over our 40-y modeling period (Fig. 2*A*).

## Supplementary Material

Supplementary File

## Data Availability

Forest inventory data, and other data used in this analysis, are available from the BioSum web portal at http://biosum.info/CEC/. All other study data are included in the article and/or *SI Appendix*.

## Change History

November 24, 2021: Figure 3 has been updated.
