# Supplementary material for: Innovative wood use can enable carbon-beneficial forest management in California
Source: Proc Natl Acad Sci U S A. 2021 Nov 22;118(49):e2019073118. doi: 10.1073/pnas.2019073118 (PMC8670525; doi:10.1073/pnas.2019073118)
Supplement: Supplementary File [file pnas.2019073118.sapp.pdf]

PNAS
www.pnas.org

Supplementary Information for

Innovative wood use can enable carbon-beneficial forest management in California

Bodie Cabiyo, Jeremy S. Fried, Brandon M. Collins, William Stewart, Jun Wong, Daniel L. Sanchez*

*Author to whom correspondence should be addressed.

Email: sanchezd@berkeley.edu

This PDF file includes:

Supplementary Methods and Results text

Figures S1 to S6

Tables S1 to S9

SI References

INTRODUCTION

In this study, we employ multiple models to understand the net carbon balance of forest treatment, wildfire, and harvested wood utilization (Table S1). These three components are described in detail in the following methods. In Section 1, we describe our approach to modeling forest management, wildfire hazard, and the carbon accounting associated with both. In Section 2, we describe our approach to lifecycle accounting of harvested wood, including the process emissions, substitution benefits, and end-of-life for several residue-based products (Table S2), as well as for conventional sawtimber products.

To understand the net carbon balance of management, we evaluate four management and wood-use scenarios which are designed to bracket a range of likely futures in California (Table S3). These scenarios represent the aggregation of our in-forest modeling, including management and wildfire, and our harvested wood accounting (i.e. "out-of-forest" modeling).

Table S1. *The three main components of this analysis rely on the combination of multiple models.*

**Component**	**Model used**	**Overview**	**Section**
Forest growth, management, and wildfire hazard	FVS, BioSum	We model six treatments in FVS over 40 years, model the economics, wildfire hazard, and wood supply for each, and then choose the optimal treatment for each plot.	1.1, 1.2
Wildfire carbon impacts	Stochastic wildfire model	We build a stochastic model to estimate wildfire occurrence based on future fire probabilities and fire weather.	1.2
Substitution benefits, process emissions, and end-of-life carbon accounting of wood products	Cradle-to-grave accounting based on several published LCA papers	We combine published lifecycle values by harmonizing system boundaries and emissions from harvest, transport, and electricity across all pathways.	2

Table S2. *Lifecycle carbon benefits for nine forest residue product pathways, in terms of tC benefit/tC in feedstock. Storage includes landfilled wood and carbon in long-lived products, but does not include storage from CCS, which is included in process emissions. Technologies included in the IWP scenario are indicated with (*).*

Residue pathway	Substitution	Process emissions	Storage	Total	Primary references
Biopower	0.13	-0.02	0.00	0.11	(1)
Decay	0.00	0.00	0.14	0.14	(2)
Biochar	0.12	-0.02	0.26	0.36	(3, 4)
Pyrolysis fuels + char	0.63	-0.34	0.14	0.43	(5)
Pyrolysis fuels	0.63	-0.20	0.00	0.44	(4–6)
Ethanol + CCS*	0.11	0.53	0.00	0.64	(7, 8)
FT fuels + CCS*	0.35	0.46	0.00	0.81	(9)
Biopower + CCS*	0.10	0.72	0.00	0.82	(1)
OSB*	0.94	-0.30	0.54	1.18	(10, 11)
GluLam*	0.94	-0.16	0.48	1.26	(11, 12)
Hydrogen + CCS*	0.80	0.85	0.00	1.65	(13, 14)

Table S3. *We combine the in-forest modeling and lifecycle accounting of wood products across four scenarios.*

Scenario	Residue utilization	Sawtimber utilization	Economic criteria
Low BAU	Biopower / Decay	Current product mix	Manage only corporate-owned land where net revenue >$2500/ha (>$1000/acre)over the modeling period
High BAU	Biopower	Current product mix	All possible management with a residue price of $0
Innovative Wood Products (IWP)	Even mix of IWP product basket	Current product mix	All possible management with a maximum residue price of $100/ODT
IWP + Housing	Same as IWP	Additional sawtimber over Low BAU is used for multi-use and multi-family buildings	Same as IWP

1 IN-FOREST CARBON

1.1 BIOSUM AND FVS MODELING APPROACH

This analysis applies the FIA BioSum modeling framework (1, 2; http://biosum.info) to understand management outcomes on California timberland. We start with data collected from 5,404 field-sampled Forest Inventory and Analysis (FIA) plots between 2005 and 2016 that represent approximately 33 million acres (13.4M ha) of California forest land. We refined this comprehensive, representative forest land sample to limit our analysis to forests offering the most promising potential for management. We retained in the dataset forested "conditions" (full or partial plots) that are classified as timberland[1] and as one of four common California forest types: mixed conifer, Douglas-fir, True fir, and Ponderosa pine. We exclude coast redwood forests, which present little fire hazard, and hardwood forests, which are rarely managed for timber. We consider only the three owner classes that account for nearly all of California's timberland: private (corporate and non-corporate) and National Forest System (NFS).

Forest growth, management, and potential fire outcomes are simulated over 40 years with the Forest Vegetation Simulator (FVS) and the associated Fire and Fuels Extension (FFE), after converting those forested conditions from the FIA database into FVS stand data. Because each "stand" comes from an FIA plot, it represents a known area of California's forest.

Subsequently, we evaluate effectiveness, and estimate costs incurred by and revenues generated from five management sequences. We use multi-criteria optimization to choose the best management sequences for each stand. Based on this optimization, BioSum calculates quantities of merchantable wood and residues[2] that could be delivered from these forests to an existing network of processing facilities. Fried, et al. 2016, upon which this work is based, explains the modeling approach for both FVS and BioSum in detail.

1.1.1 Management Sequences and FVS

We simulate five management sequences (Table S4) designed to represent forest restoration-motivated thinning regimes compatible with provisions of the Sierra-Nevada Forest Plan. Sequences are defined as a repeated treatment over the modeling period. Treatments can occur only once or twice over the 40-year modeling period under rules that set the minimum re-treatment interval at 20 years. The treatments differ with respect to thinning style, maximum size of trees allowed to be harvested, and approach to addressing surface fuels. Each treatment considers a basal area reduction of up to 33% and implements thinning with a whole-tree harvest system, using either a mechanical harvester (on gentle slopes) or manual (chainsaw-based) felling (on steep slopes), so harvested trees of merchantable size generate very little surface fuel[3]. After thinning, surface fuels are either reduced by prescribed fire or rearranged via lop and scatter (severing and scattering stems near where they're cut). We also modeled

[1] Land capable of producing an average of at least 20 ft^3/acre/year (0.56 m^3) of wood and not legally reserved from timber management.

[2] Inclusive of branches, bark and foliage.

[3] However, on both gentle and steep slopes, trees 51 cm DBH and larger are assumed to require manual felling and bucking with chainsaws, so where such trees are cut, they contribute "activity fuels".

a “Grow Only” sequence to represent the hands-off approach currently typical on most publicly owned forestland.

Table S4. *Management sequences modeled. Thinning styles are thinning proportionally across diameter classes (“ThinDBH”) and thinning from below (“ThinBBA”). Entry threshold is the basal area that triggers thinning (provided there is a 20-yr hiatus between thinning entries). Max DBH is the breast height diameter of the largest tree allowed to be harvested as part of the thinning, on public (pub) and private (pvt) land. Surface fuel method is either rearrangement (lop and scatter) or reduction (via prescribed fire).*

**Thinning Style**	**Entry Threshold (m^2)**	**Max DBH (cm) cut, by ownership**	**Surface fuels treatment**
ThinDBH	BA≥11	91 pvt/76 pub	Rx Fire
ThinDBH	BA≥11	91 pvt/76 pub	Lop/scatter
ThinDBH	BA≥11	91 pvt/53 pub	Lop/scatter
ThinBBA	BA≥11	91 pvt/76 pub	Lop/scatter
ThinBBA	BA≥11	91 pvt/76 pub	Rx Fire
GrowOnly	NA	NA	NA

The modeling approach used in FVS is described in detail in (17).

1.1.2 Management Optimization with BioSum

FVS simulation of forest management sequences, described above, is one part of the analysis workflow supported by the BioSum modeling framework. BioSum’s four major modules include: (a) **Database**, for loading and managing forest inventory data from the FIADB, the national FIA database; (b) **FVS**, for creating FVS input files from the FIA database, defining prescriptions and management sequences, and importing FVS outputs to drive later stages of the analysis; (c) **Processor**, which calculates harvest costs and revenue from sales of harvested wood for each combination of stand and sequence, from “cut list” data output from FVS, and (d) **Optimizer**, where the analyst sets criteria for what constitutes successful management outcomes, chooses one or more attributes to optimize, subject to constraints involving other objectives and/or economic feasibility, and establishes rules to break ties among alternative sequences that achieve the same optimum to arrive at a single, optimal management sequence for each stand. Extensive documentation on BioSum and examples of its use are available at http://biosum.info.

1.1.2.1 Modeling management costs and revenues

We define management sequences to allow thinning entries in each stand to occur in up to two of four years over a 40-yr time horizon: years 1, 11, 21, and 31. When basal area exceeds a prescription’s threshold and the rule that separates thinning entries by 20 years is satisfied, FVS simulates the harvest activity and resulting changes to stand characteristics and BioSum calculates associated management costs and revenues. Fixed costs of moving harvesting equipment to a treatment site are distributed over an assumed 40-acre (16-ha) harvest area. The 16% of stands derived from FIA plots that are more than 2500 ft. (760 m) from an existing forest road (surfaced or unsurfaced, excluding skid trails) are assumed to be inaccessible and ineligible for management, as modeling construction of new access roads is beyond the scope of a BioSum analysis.

Delivered merchantable (sawtimber) wood values are set according to the California Board of Equalization timber tax reports (18). Residue prices are set at maximum price of $100 per ODT delivered ($50 per green ton). Trees 20 cm DBH and larger are processed and valued as merchantable saw logs. Small (10 – 20 cm DBH) harvested trees are assumed to be chipped (boles and branches), along with trees with at least 50% of bole volume classified as cull and non-commercial species (mainly hardwoods) of all sizes. Trees less than 10 cm DBH are cut, lopped (cut in half), and scattered near where they are felled, except on steep slopes where this threshold is 13 cm. Tree harvest systems and associated costs are dependent on plot slope. We classify “steep” slopes as 40% grade (18 degrees) or more. On steep slopes, we model treatment cost using more expensive harvest systems (cable manual whole tree).

To account for the costs of moving both saw logs and wood residues from the forest to processing facilities, BioSum simulates road travel time from the point on the road network nearest to each FIA plot to each potential processing site. Travel time is determined by rated road speed and the locations of existing processing facilities. BioSum identifies the closest (in time) merchantable facility and bioenergy facility for each plot and assumes that harvested wood would be transported only to those facilities. We use a list of 36 active and idle wood-processing facilities, and assume these will be scaled-up, restored, and/or supplemented with additional, newly constructed, co-located facilities in response to increased wood supply. We assume a round-trip haul cost of $7 per green ton-hour for both types of wood. For each management sequence and stand, these transportation costs are combined with harvest costs and wood revenues to calculate the net revenue expected to result from management in a given decade. These can be summed over all decades in which treatment occurs to obtain a 40-year net revenue estimate.

1.1.2.2 Low-value feedstock suitability

About one fifth of the forest residue wood basket in IWP is composed of small trees (10-20 cm), and the rest is composed of branches and tops of larger trees, as well as the entirety of non-merchantable trees (mostly hardwoods). While we model these residues as an aggregate supply of chipped material (i.e. inclusive of boles, branches, bark, and foliage), we assume that this material could be sorted at the landing site or a receiving facility. For example, sorting at the landing site could involve stripping branches off treetops and small trees to produce boles suitable for OSB stranding (19) and dirty biomass chips suitable for biofuels or biopower. Sorting at the receiving facility could involve filtering fines and bark for use as process heat and chips as core material in three-layer OSB (20). While these sorting processes are not directly modeled here, we expect that they would not substantially change the cost of delivered feedstock because the haul cost, which makes up the largest portion of the total delivered cost for these materials, remain unchanged given that the costs of their felling and yarding are already accounted in the cost of merchantable wood. In some cases, it may not be possible to sort low-value wood because of economic constraints or insufficient quantity. The ability to tolerate bark and needles for innovative wood products is process specific. However, we are confident that numerous technologies can accommodate dirty chips, including biofuel production technologies. Modern gasification technologies, which underlie many of the biofuel technologies we consider here (including hydrogen), are tolerant of varying wood quality. For instance, Red Rock Biofuels, a Fischer-Tropsch diesel biofuel facility under construction in Lakeview, OR, will accept “dirty” chips including bark and needles. In California, a recent budget proposal by the Department of Conservation included $50M for a 30,000-ODT/year forest residue-to-fuels gasification facility. Further, there are several examples of commercial gasification technologies that process municipal solid waste, a feedstock with similar

heterogeneity to biomass chips. Examples include gasifiers developed by Omni Conversion Technologies and multiple gasification technologies currently operational in Canada (21). We model a suite of technologies to account for the fact that no single technology can be expected to utilize all the low-value wood produced on California timberland.

1.1.2.3 Management decision criteria

We set three sequential optimization criteria to select the optimal management sequence for each stand in BioSum. First, all combinations of stand and management sequence that were incapable of generating positive net revenues were dropped from further consideration. The second criteria, which defines treatment effectiveness, is defined as a reduction in the 40-yr mean fire-induced mortality, as a fraction of total live basal area, predicted by FFE-FVS under severe fire weather conditions relative to the same hazard metric calculated for the Grow Only sequence (see Section 1.2, below). Finally, the optimal sequence was defined as the effective sequence that maximized live tree carbon at the end of the 40-year analysis period. In cases where two or more effective management sequences had the highest 40-year live tree carbon, the sequence with the greatest reduction in fire mortality (as defined above) was chosen.

1.2 Wildfire Modeling

We model potential fire outcomes for each stand, year, and treatment sequence with the FVS Fire and Fuels Extension (FFE). These potential fire outcomes are modeled independently for each year and represent “what-if” fire hazard metrics. We develop a stochastic model to understand how these potential outcomes would manifest under a realistic fire regime, since only a small portion of the forest will burn in any given year. We run 5000 Monte Carlo simulations to reflect the inherent spatial and temporal variability in wildfire. In each simulation, we randomize (a) how many plots burn, (b) which plots burn, and (c) when they burn. In the first step, we randomly select a predicted fire frequency from a log-normal distribution. We assume a 3.8% increase (22) over an observed annual fire probability of 0.089% (23), so our projected annual fire probability is 0.092%. We assume this new frequency varies by the same amount (i.e. the standard deviation is 3.8%). These assumptions are within the bounds of the modeling conducted by Mann et al. (2016) for the 2030-2050 time period. Subsequently, we randomly select a cohort of plots that will burn over a forty-year period and then randomly select the year in which they burn. Predicting future wildfire occurrence and extent is inherently problematic, but a multi-step approach to modeling spatial and temporal stochasticity can improve accuracy of models (24).

1.2.1 Combustion, Post-Fire Decay, and Reduced Growth of Fire-Affected Stands

We consider three primary fire effects: combustion emissions, post-fire decay of fire-killed trees and reductions in stand growth.

1.2.1.1 Combustion

We model combusted carbon as:

$$C_{combusted} = C_{FWD} + C_{CWD} * F_{CWD} + C_{LT} * F_{LT}$$

Where the total carbon emitted in a wildfire event is the sum of fine woody debris carbon (FWD), coarse woody debris carbon (CWD) and live tree carbon (LT) times the fraction (F_{CWD} and F_{LT}) of aboveground carbon expected to combust. We assume fine woody debris combusts completely. We parameterize $F_{combustion}$ with values observed in comparable dry conifer forests in southern Oregon (25). We apply

$F_{combustion}$ values that are specific to both wood class (CWD and LT) and wildfire basal area mortality class (low: <20%, moderate: 20-95%, severe: >95%). For most fires (i.e. "moderate" mortality), $F_{combustion}$ for live trees is 0.07. The values for $F_{combustion}$ used here are very low compared to values commonly used in combustion models but are likely a better representation of observed combustion rates (26). For comparison, we estimate $F_{combustion}$ for CWD and LT combined at 0.15 based on a study of pre- and post-fire observations on FIA plots across California, although the exact value is not specified in the study (27). While this combustion rate increases the magnitude of combustion emissions from wildfire, thus improving the carbon benefits of management, it does not change the core findings of our scenario analysis.

1.2.1.2 Post-Fire Decay

We assume a post-fire decay rate constant of 0.016 yr^{-1}, which is based on observed decomposition rates in similar dry, coniferous forest after a variable-severity fire (28). We take this decay rate to be representative for the species and environmental conditions in the present study. Decomposition rates vary depending on climate, species, char-content, and whether dead wood remains standing or falls to the forest floor. We do not explicitly model snags (standing dead wood), although the decay rate of snags is similar to our generalized rate (28). The decay rate constant we use here is specific to post-fire decay, which few studies have quantified. This rate is lower than reported rates for non-post-fire decay. For example, Douglas fir, the most common species in our study, has a CWD decay rate of 0.021 in California (2). Using a low rate constant limits the magnitude of wildfire-induced emissions in our simulations, largely because we consider a 40-year modeling period. When we test a decay rate of 0.033 yr^{-1} (i.e. a half-life of 20 years), we find that decay emissions roughly double, increasing the magnitude of carbon benefit associated with management. This does not, however, change the core conclusions of our scenario analysis. We assume simple exponential decay across all wood classes. Although some models suggest a two-stage decay equation to capture variable rates across the decay process, this effect is poorly defined in the literature and likely to be small (29).

1.2.1.3 Post-Fire Growth Adjustment

After a fire occurs in a stand, we reduce the total live tree carbon and future growth of the stand proportionally to the basal area mortality. We model post-fire live tree carbon during any given year *i* after a fire (LTC_i) with the following equation:

$$LTC_i = LTC_{0i} * (1 - BA_{mortality})$$

Where LTC_0 is the FVS-modeled live tree carbon during year *i*, absent fire, and $BA_{mortality}$ is the modeled basal area mortality fraction during the year of the fire. While basal area mortality is not always a perfect proxy for mortality volume (and thereby carbon), in our dataset of over 300,000 FVS observations of predicted annual stand-level wildfire effects, we find a Pearson correlation coefficient of 0.96 between ($BA_{mortality}$ * live tree volume) and FVS-reported mortality by volume. In the case of fires with very high $BA_{mortality}$ (>95%) on corporately managed land, we assume that forests grow 50% faster than the base case to account for actively managed regeneration (30), although this approach likely underestimates regrowth on these stands. At the high end of this effect, stands with 95% mortality will regrow at a rate of 7.5% of their pre-fire growth rate, including the 5% of BA (often large trees) that was not killed. The effect of this assumption is very small during our 40-year modeling period. We also assume zero decay of merchantable-sized trees after fire in these forests, because rapid salvage logging is common in corporately owned forests.

1.2.2 Fire Weather Definitions

Fire weather parameters are required for FVS-FFE to provide estimates of potential fire behaviour under different fire weather conditions. All of the forests we modeled in this study using the NC, WS, CA and SO FVS variants are considered to be in arid or semi-arid climate types (31). Fire weather for 90th and 97.5^{th} percentile conditions, derived from 30 years of Remote Access Weather Station (RAWS) data from multiple locations, filtered for the fire season, was analysed with Fire Family Plus to generate parameters supplied to FVS-FFE. We relied on the 97.5^{th} percentile and 90th percentile weather parameters to represent a range of likely future fire weather conditions. Temperature, wind speed, and relative humidity assumptions are given in Table S5.

Table S5. *Weather Parameters Used to Model Fire in Conifer Forests of the Sierra and Interior Coast Ranges*

Parameter	97.5^{th}% Weather	90th% Weather
Wind speed, km/h (mph)	32 (20)	26 (16)
Temperature, °C (°F)	33 (91)	32 (90)
Relative humidity (%)	15	17

Assumed fuel moisture parameters (Table S6) are similar to those used by others who have modeled fire potential in California forests, and are assumed constant over the 4 decades of FVS projection. For example, 1-, 10- and 100-hour, live herb and live woody fuel moistures and wind speed data are similar to those reported by (32–36). 1000-hr and duff moisture parameters are similar to the observations reported in (37, 38) and are appropriate for Sierra, Cascade, and interior coast range coniferous forests. It is possible that the effect of this assumption will tend to understate severity of fire and mortality rate if climate change increases temperature and wind speed and/or reduces humidity.

In the Results, we present a mean of wildfire simulation results conducted with 97.5 and 90th percentile fire weather. While these weather scenarios represent more extreme conditions, they reflect both the observed tendency that a majority of fire area burns in a relatively small number of very large fires, which occur under more extreme fire weather (24, 39), and the likelihood of increasing incidence of severe fire weather within our modeling period (40). Several recent studies have used similar increased incidence of severe fire weather in their future projections (41–44).

Table S6. Fuel Moisture Parameters Used to Model Fire in Conifer Forests of the Sierra and Interior Coast Ranges

Fuel Type	Description	97.5th% Weather Conditions	90th% Weather Conditions
1-hour fuel	The 1-h time lag fuel pool consists of dead and down fuel particles less than ¼-inch (6 mm) in diameter (i.e. litter).	1.8	3
10-hour fuel	The 10-h time lag fuel pool consists of dead and down fuel particles between ¼-inch (6 mm) and 1-inch (25 mm) in diameter.	2.3	3.7
100-hour fuel	The 100-h time lag fuel pool consists of dead and down fuel particles between 1-inch (25 mm) and 3 inches (75 mm) in diameter.	4.2	6.6
3" fuel	The 1000-h time lag fuel pool consisting of down fuel particles larger than 3 inch (75 mm) diameter.	8	12
Duff	Duff	20	40
Live woody	The live woody fuel pool is the foliage of shrubs and small trees plus the fine live branch wood of shrubs and small trees. Fine live branch wood is generally considered branches less than ¼-inch (6 mm) in diameter.	70	80
Live herb	The herbaceous fuel pool is the load of standing live and dead grass stems and other herbaceous fuel. Both the live and dead standing components are included in this fuel pool; the live and dead components are separated at the time of fire behaviour simulation	30	30

2 WOOD PRODUCTS LIFECYCLE ACCOUNTING

2.1 STRUCTURAL WOOD PRODUCTS MODELING

We use a harvest-to-grave system boundary for the lifecycle accounting of merchantable wood products over 40 years. We consider one ton of harvested carbon as the primary unit of analysis. We model the in-forest carbon outcomes from increased management as previously described. The methods used to calculate product substitution and end-of-life are described below.

2.1.1 Substitution and Production Emissions

To model the substitution benefits attributable to the use of merchantable wood, we adapt the methodology of (11) to the California market context. Smyth et al. (2017) calculate an economy-wide displacement factor for wood products in construction using published values for emissions from extraction, transportation, and production for common building materials. Here, we retain all values used by Smyth except for end uses for wood products, which are economy-specific. In place of the Canada-specific values used in Smyth et al. (2017), we use historical California HWP end use data (45). Because the end use categories reported by Smyth et al. (2017) and by Christensen et al. (2017) are different, we aggregate the categories used by Christensen et al. into the less granular categories used by Smyth et al. (Table S7). As in Smyth et al. (2017), here we disregard two end-use categories: packaging/shipping materials and non-disclosed end uses (e.g. home-made furniture). Christensen et al. aggregate saw timber biomass (e.g. sawdust) and biomass chips, so here we assume that 5.2% of the merchantable yield goes towards biopower and 94.8% goes towards wood products (46). Using this approach, we calculate the substitution benefit of harvested merchantable wood to be 0.75 tC/tC, which is within the range of estimates for other regions (11, 47).

We also consider an alternative wood end use scenario in which 100% of increased wood supply is used to displace steel and concrete buildings (Table S7). As a result, a larger fraction of wood is directed towards the new, multi-family and multi-use building categories. In this scenario, the substitution benefit is 1.75 tC/tC. While this scenario may be in part achieved through increased production of mass timber products (e.g. Cross-Laminated Timber), we do not explicitly model those products here.

Table S7. *Saw timber end use ratios and their associated half-lives.*

Christensen et al. (2019) category	Smyth et al. (2017) category	Half-life from Skog (2008)	End-use fractions of wood used	Housing Scenario end-use fractions
New housing, single family	Single family	80	0.18	0.06
New housing, multi-family	Multi-family	50	0.2	0.34
New non-residential	Multi-use	30	0.13	0.38
Residential remodel	Flooring	26	0.34	0.12
Manufacturing	Furniture	30	0.15	0.05
Other industrial products	Decking	30	0.17	0.06
**Total**			**1.00**	**1.00**

2.1.2 End of Life and Embodied Carbon

To model the lifecycle of embodied carbon in HWP, we calculate a category-weighted mean half-life from primary wood product half-lives defined in (48) for the United States of 38 years (Table 4). After a wood product's usage, we assume that 65% of all post-consumer residues (retired wood products) are sent to landfills, 25% to bioenergy facilities, and 10% are not collected (49). Of all carbon in post-consumer residues sent to landfills, we assume that 90% is permanently inert, and we conservatively assume that decay of the remaining 10% happens instantaneously (50). We assume that post-consumer residues generate electricity with a lower heating value of 13.9 GJ/ODT and a heat rate of 80 kWh/mmBtu (76 kWh/GJ) (51), and that the bioenergy produced displaces grid electricity with a carbon intensity of 225 gCO2e/kWh (52). In sum, we consider the downstream storage in use, product substitution benefits, and end-of-life, including post-consumer residue bioenergy generation, fossil electricity substitution and carbon permanently sequestered in landfills.

2.2 Forest Residues Lifecycle Accounting and Economics

As with saw timber, we use a harvest-to-grave system boundary for the lifecycle accounting of forest residue products over 40 years. We include harvest and transport, production emissions, product substitution, and end-of-life. Biogenic (in-forest) carbon accounting is described in Section 1. We consider one ton of harvested carbon as the primary unit of analysis. The assumptions and methods for each product are described below. We aggregate values from several published Lifecycle Assessments (LCA's) and adjust those values where necessary to achieve consistency. For every product, we rely on LCA's that have either a wells-to-wheels or cradle-to-grave system boundary. We normalize harvest and transport emissions for all products to be consistent with values used in The Greenhouse Gases, Regulated Emissions, and Energy Use in Transportation (GREET) model (9). Where necessary, we adjust values so that forest residues are carbon neutral, because we account for in-forest carbon changes in Section 1. We assume a travel distance of 145 km (90 mi) with backhaul. We assume all electricity to have a California-average carbon intensity of 225 gCO_2e/kWh (52). Cumulative carbon benefits for each product pathway are given in Table S3.

To roughly estimate potential delivered forest residue prices, we assess the internal rate of return for several innovative wood products under a range of delivered residue prices in California over a 20-year financial period. We assume a fuel price of $2/GGE (gallon of gasoline equivalent) and an LCFS credit price of $100/tCO2 abated. We assume $224 / MSF (3/8" basis) for oriented strand board, and no additional policy support. We derive our cost and performance assumptions from existing techno-economic analyses of large-scale production (5, 19, 53, 54). Figure S6 shows the internal rate of return for each of these products for varying delivered residue prices. Based on this analysis, we expect innovative wood products to return positive returns for delivered wood prices as high as $100/ODT. This value is higher than current market rates, but similar to what has been modeled in previous work (14, 55, 56).

2.2.1 Lignocellulosic ethanol with CCS

For the LCA of lignocellulosic ethanol production with carbon capture and storage (CCS) from forest residue we rely on modeling done by McKechnie et al. (2011) and Liu et al. (2011) (7, 8). We obtain relevant process information about forest biomass harvesting and operations from McKechnie et al. (2011), and fuels production with CCS from Liu et al. (2011). We analyze an E85 (85% ethanol, 15% gasoline) pathway from forest biomass, which allows for more direct substitution of gasoline relative to E100. To account for the efficiency loss when switching from conventional gasoline to E85, we assume an efficiency of 5 km/L for E85 and 7.69 km/L for gasoline (7). McKechnie et al. (2011) also include a coproduct credit from natural gas-fired sources for electricity, which we modify to assume displacement of average California grid electricity in 2016 (225 gCO_2e/kWh).

2.2.2 Fischer-Tropsch diesel with CCS

For the LCA of Fischer-Tropsch (FT) liquids production with CCS from forest residue, we rely on Xie et al. (2011) (9), who document various combinations of feedstocks for FT liquids generation, including 100% forest biomass. Xie et al. (2011) use GREET for their LCA using a well-to-wheels boundary: from biomass collection to tailpipe emissions. Forest residues are assumed to have an LHV of 13.243 mmBtu/ODT (13.9 GJ/ODT) and the FT process has a 0.5 LHV efficiency, with 92% are FT liquids and 8% is electricity by LHV. In their BTL-CCS case, they assume 89.9% CO_2 capture ratio with a recycling design, the well-to-wheels emission factor is -150 $kgCO_2e/mmBtu$ (-142 gCO_2/MJ). Xie et al. (2011) assume that the

coproduced electricity displaces the average US grid electricity in 2009 (554 gCO_2e/kWh). We update this assumption so that the co-produced electricity displaces the average California grid electricity in 2016. We also update the forest operation and transportation emissions, using data from (7) as discussed above.

While the FT process produces a mixture of diesel and gasoline, we assume a constant baseline carbon intensity (CI) of 100.45 gCO_2e/MJ (diesel's CI, as compared to 100.82 gCO_2e/MJ for gasoline) for all FT liquids produced since Xie et al. (2011) do not report a breakdown by fuel type.

2.2.3 Biochar production

The LCA of biochar production and use from forest residues relies on data from Roberts et al. (2010) and Woolf et al. (2010) (3, 4). Roberts et al. (2010) analyze feedstocks most similar to the forest residues modeled here, and Woolf et al. (2010) provide general characteristics of biochar. We assume that biochar is produced from the slow pyrolysis of forest residues.

Roberts et al. (2010) use a cradle-to-grave system boundary, which begins from feedstock handling to carbon sequestered by biochar. We assume that the char yield is 29.6% (by weight) of the feedstock input in a slow pyrolysis process. The net stable C in char is 574 $kgCO_2e$/ton feedstock, which includes emissions from pyrolysis. Roberts et al. (2010) assumes that transport and residue collection emits 19 $kgCO_2e$/ ton feedstock, but we modify this assumption to be consistent with other pathways. Lastly, Roberts et al. (2010) estimate a natural gas substitution benefit of 229 $kgCO_2e$/ton feedstock. For end-of-life, we assume that the biochar has a labile fraction of 15% with a half-life of 20 years and a recalcitrant fraction of 85% with a half-life of 300 years (4).

2.2.4 Biopower with and without CCS

The LCA of the electricity with CCS pathway using forest residue feedstock relies on data from Sanchez et al. (2015) and Xie et al. (2011) (1, 9). While Sanchez et al. (2015) consider electricity generation from a blend of lignocellulosic biomass, we supplement this with the LHV of forest residues from Xie et al. (2011) to be consistent across scenarios. We use the biomass integrated gasification combined-cycle (IGCC) with CCS scenario in Sanchez et al. (2015).

Sanchez et al. (2015) consider the growth of the biomass until the generation of the electricity and the subsequent CO_2 storage to be its system boundary. Since forest carbon is accounted previously in our model, we remove the agricultural phase (0.004 tCO_2/mmBtu (3.8 gCO_2/MJ)) from the total carbon intensity (-0.0802 tCO_2/mmBtu (-76 gCO_2/MJ)). Instead of an LHV of 17 mmBtu/ODT (18 GJ/ODT), we assume 13.2 mmBtu/ODT (13.8 GJ/ODT) from Xie et al. (2011) to be consistent with the Fischer-Tropsch diesel pathway. We use a heat rate of 16.32 mmBtu/MWh, implying a facility with a 21% efficiency. For the biopower without CCS pathway, we use a heat rate of 12.5 mmBtu/MWh (13.1 MJ/kWh) (1). We also assume that the generated electricity displaces the average California grid electricity in 2016. We update the transportation assumptions in Sanchez et al. (2015) to our common assumptions, given above.

2.2.5 Pyrolysis fuels, with and without biochar co-production

The LCA of biofuels production and use from forest residues relies on Li et al. (2017), who present a techno-economic assessment of a 2000 t/day facility with red-oak (*Q. rubra)* feedstock (5). This facility produces 50.73 gallons (192L) of gasoline/ODT feedstock and 37.01 gallons (140L) of diesel/ODT, which we assume replace conventional gasoline and diesel. The facility burns the non-condensable gas and

biochar for process heat, but natural gas and electricity are also used for the bio-oil stabilization process. This process has a reported carbon intensity of 31.8 gCO_2e/MJ (5). The non-condensable gas and biochar burnt for process heat is assumed to displace natural gas.

We model an alternative process in which biochar is reserved. Pyrolysis of loblolly pine residue yields 50.7 (wt%) bio-oil, 10% char, and 25.3% non-condensable gas (6). Meanwhile, the facility still produces the same amount of gasoline and diesel as above. Li et al. (2017) use an HHV of biochar of 23.05 MJ/kg, and we assume for this to be the same for the biochar that is produced from loblolly pine instead of red oak. We calculate the energy produced from combustion of biochar and replace it with the equivalent amount of energy from natural gas combustion. We use a natural gas carbon intensity of 50 gCO_2/MJ (1). The reserved biochar is assumed to be the same as described by Woolf et al. (2010), although it may have different properties given that it is produced via fast pyrolysis instead of slow pyrolysis.

2.2.6 Hydrogen production

For hydrogen production, we rely on the LCA conducted by Antonini et al. (2021) of hydrogen gas produced from wood waste (13). We model their entrained flow gasifier with pre-combustion CO_2 capture and storage, which has a CI of -130 gCO_2/MJ. This process was chosen because it has the highest rate of carbon capture amongst all modeled hydrogen production processes. We adjust this CI to account for using California grid electricity, which has a lower CI than the EU grid used in their analysis (400 gCO_2/kWh). We further adjust this CI to include harvest operations and transportation to a processing facility, consistent with our other pathways. To convert the functional unit from MJ to ODT-feedstock, we use an energy conversion efficiency of 70%. To model substitution benefits, we assume this hydrogen displaces conventional hydrogen produced from natural gas via steam reforming in California, which has a carbon intensity of 120 g/MJ (14).

2.2.7 Oriented Strand Board (OSB) and GluLam production

We rely on Puettmann et al. (2013) to represent the lifecycle greenhouse gas emissions from oriented strand board (OSB) production (10). Puettmann et al. (2013) use a well-to-wheel approach to assess the lifecycle impacts of producing OSB in the Southeastern United States with loblolly pine (*P. taeda L.)* and slash pine *(P. elliottii E.)* feedstock. Puettmann et al. (2013) assume a transportation distance for feedstock of 89 miles, consistent with our other pathways. We modify their calculated production emissions (275 kg CO_2e/m^3) to account for a lower carbon intensity for California grid electricity. CO2e/MWh, and an averaged Southeast United States grid carbon intensity of 454 gCO2e/kWh (52). Puettmann et al. (2013) assume wood comes from intensively managed plantations, so we instead use the carbon intensity from forest operations (e.g. harvesting) described above. We assumed a density of 614 kg / m^3 of OSB, resins excluded (10). For substitution benefits and end-of-life, we make the same assumptions for OSB as for sawtimber products.

To model the production emissions for glue-laminated beams (GluLam) we rely on the LCA conducted by Bowers et al. (2017) (12). We use values associated with their Pacific Northwest facility because the wood species mix is most similar to California (true fir species and Douglas fir (*Pseudotsuga menziesii*)). We make three adjustments to the values they report. First, Bowers et al. (2017) assume electricity comes from the WECC in 2008, which was reported as 432g/kWh (52). We update this grid carbon intensity for the California grid. Second, we adjust the sawmill efficiencies for lamstock production used (50%) to be consistent with California sawmill efficiencies (75%) (49). Third, we replace the harvest and transport-to-mill emissions reported by Bowers et al. (2017) with GREET values, consistent with our

other pathways. We assume mill residues are combusted for biopower, like our other structural wood product pathways. For substitution benefits and end-of-life, we make the same assumptions for GluLam as for sawtimber products.

Table S8*. Characteristics of engineered structural wood products. Sources include* (10, 12, 19)*, as well as Nordic Structures (GluLam feedstock).*
**CLT is not modeled in this paper but is included here for comparison.*

**Engineered wood product**	**Adhesive fraction (by mass)**	**Capital costs**	**Feedstocks**
OSB	4%	$148-166M	Small boles (e.g. pulpwood), minimum top size ~8 cm
GluLam	0.46-1.22%	Unknown; like CLT	Range; can use small boles w/ average diameter of 11 cm
CLT*	<1%	$17M	Dimensional lumber

3 SUPPLEMENTARY RESULTS

3.1 WOOD AVAILABILITY

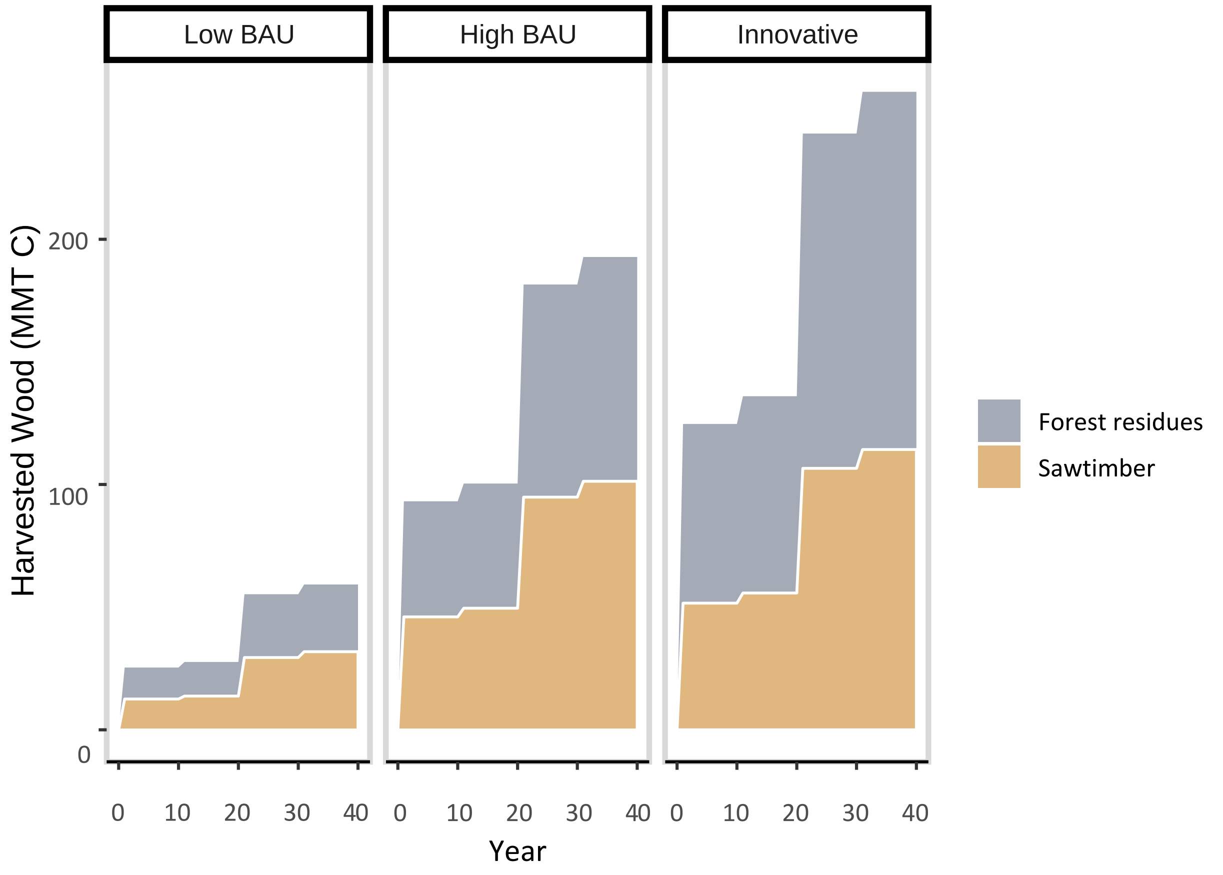

Figure S1. Cumulative harvested wood for three scenarios over 40 years, by wood class. Harvest timing is a function of both technical potential and policy constraints, including a 20-year enforced hiatus between harvest events. In Low BAU, we model management only on corporate land, where potentially profitable (net revenue >$2500/ha). In High BAU, we model management wherever it is net revenue positive with a delivered residue price of $0. In Innovative (IWP), we model management wherever it is net revenue positive with a delivered residue price of $100/ODT.

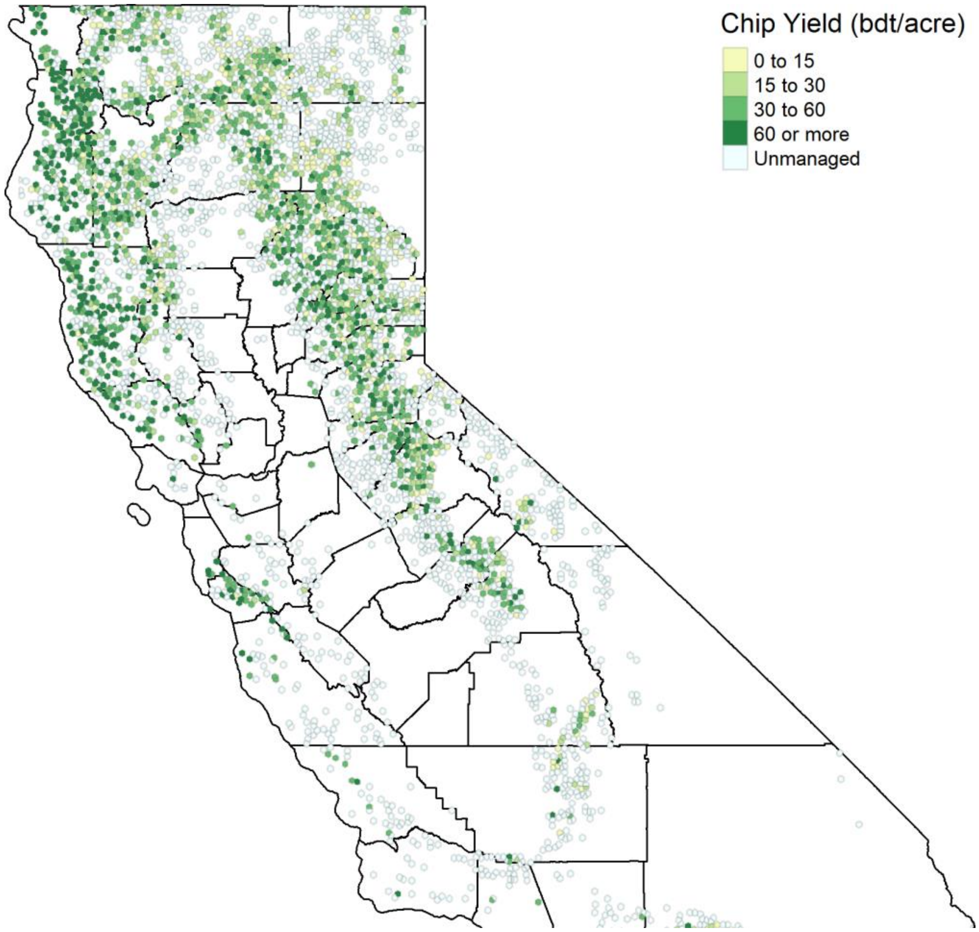

Figure S2. Forest residues produced over 40 years of management under the IWP scenario, with county boundaries shown. Each hexagon represents a single FIA plot, which is statistically representative of a larger area of forest (usually, ~2000-2500 ha).

3.2 Wildfire Modeling

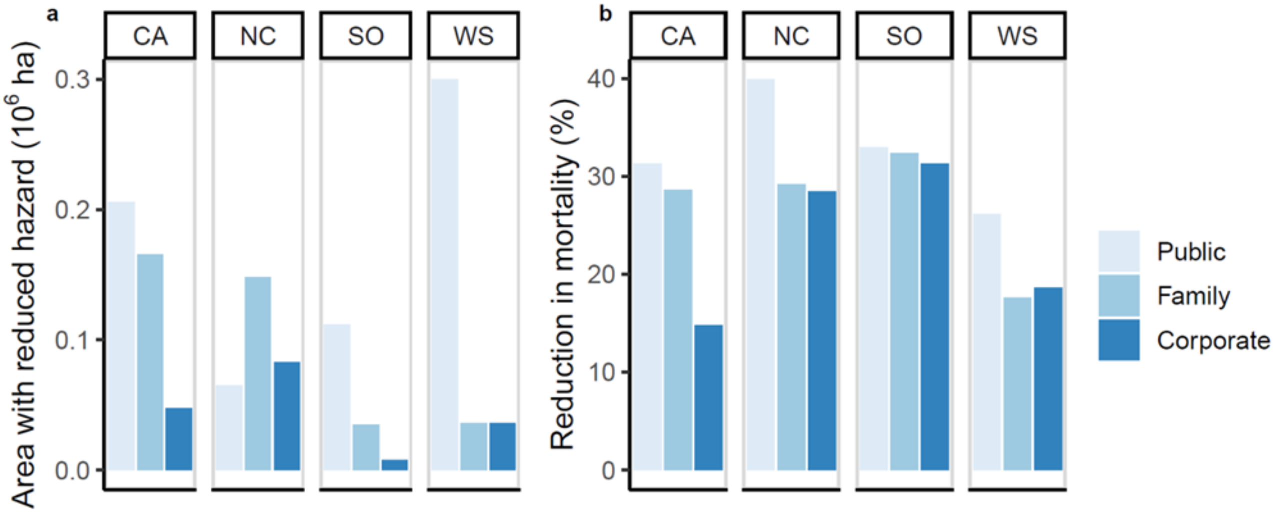

Figure S3. Reduction in stand replacing fire hazard by (a) area on which stand replacing fire may be avoided as a result of treatments and (b) mean reduction in predicted mortality under severe fire conditions in those stands (see SI Methods). Predicted mortality reduction is the difference between the percent of basal area that would die with and without treatment. Values are grouped by FVS Variants, where CA is Central California, NC is North Coast, SO is Northeast California, and WS is Western Sierra. Colors represent ownership groups, where 'Family' is non-corporate private land.

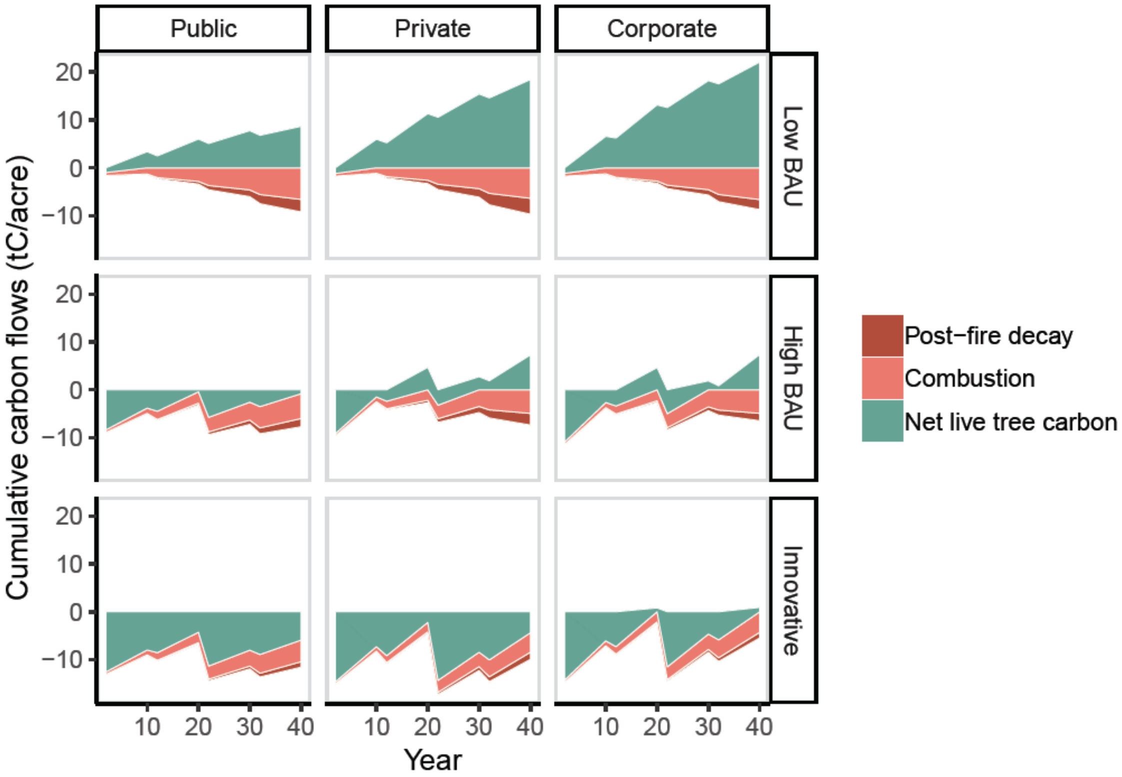

Figure S4. Cumulative in-forest carbon changes over 40 years across three scenarios and three owner groups. Net live tree carbon values are relative to carbon stocks in year zero, so negative values represent a carbon loss relative to year zero. Large changes in live tree carbon represent harvest events. In Low BAU, we model management only on corporate land, where potentially profitable (net revenue > $2500/ha). In High BAU, we model management wherever it is net revenue positive with a delivered residue price of $0. In Innovative (IWP), we model management wherever it is net revenue positive with a delivered residue price of $100/ODT. Treatment area under IWP defines the study area for High and Low BAU, which include untreated forest.

3.3 NET CLIMATE OUTCOMES, TECHNOECONOMIC ANALYSIS, AND ALTERNATIVE BAU

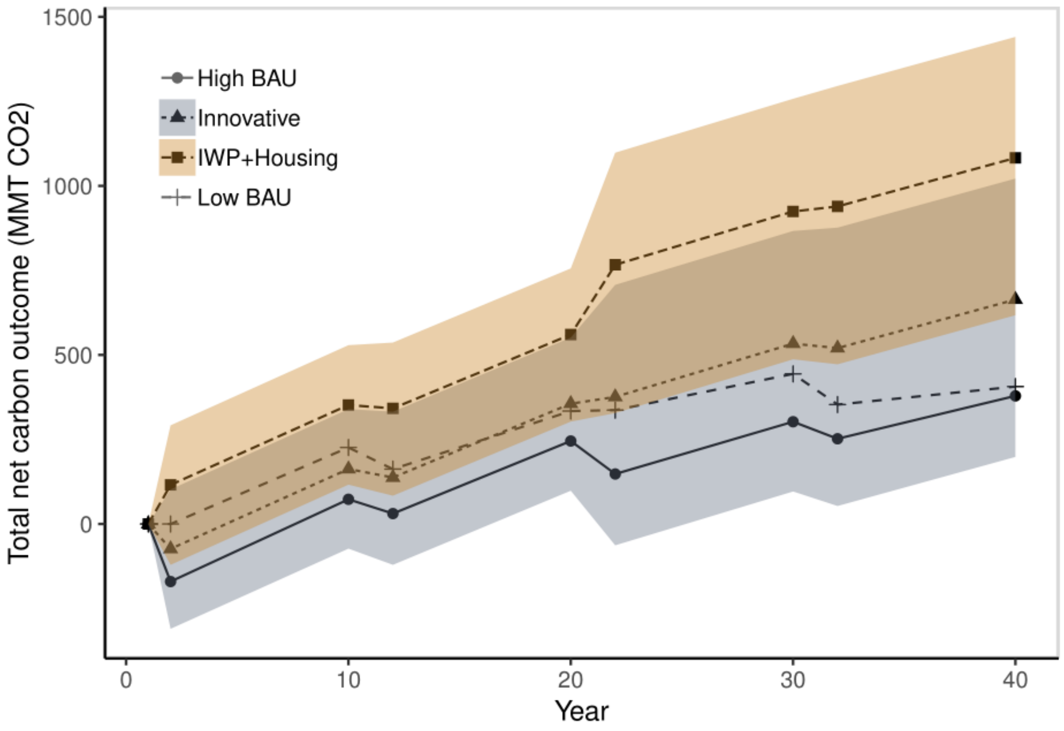

Figure S5. Net climate benefits for four scenarios, including changes to in-forest carbon and benefits from wood products. Shading represents the sensitivity of the two innovative scenarios to residue product choice. The minimum and maximum values represent the scenarios where all forest residues go to the product with the worst or best carbon outcomes, respectively, of all products examined (i.e. biopower or hydrogen + CCS). In Low BAU, we model management only on corporate land, where potentially profitable (net revenue > $2500/ha). In High BAU, we model management wherever it is net revenue positive with a delivered residue price of $0. In Innovative (IWP), we model management wherever it is net revenue positive with a delivered residue price of $100/ODT. In IWP+Housing, we assume additional sawtimber produced (over Low BAU) is used in multi-use and multi-family buildings.

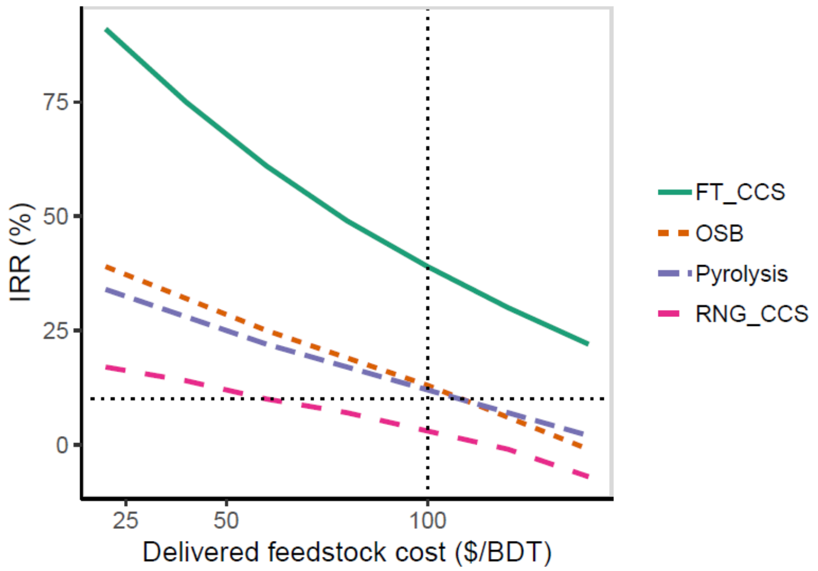

Figure S6. Technoeconomic assessment of select innovative wood technologies. Dotted lines represent the intersection of $100/ODT and an IRR of 10% (SI Methods). OSB is Oriented Strand Board. FT_CCS and RNG_CCS are Fischer-Tropsch and Renewable Natural Gas fuels with Carbon Capture and Storage.

Table S9. Mean harvested sawtimber flow and total area managed over 40 years by owner group in BAU-2.

Owner	Timber harvested (M ODT/yr)	Area managed (M ha)
Public	0.2	0.08
Corporate	0.9	0.65
Family	0.2	0.12

We investigate an alternative formulation of BAU, “BAU-2”, with better representation of management on public and family forests. In BAU-2, we model active management on all lands where a net revenue of >$12,500/ha is possible without revenue from forest residues. This threshold yields timber volumes from public and private land that approximate those reported in (Christensen et al. 2019) for 2017 (Table S9). Under this management scenario, 1.3M ODT, or 1.4B board feet (BF), per year of saw timber are harvested over the next 40 years, on average. Comparing IWP to BAU-2, we find a net climate benefit of 6.4M tCO_2 per year when considering impacts from management, wildfire, carbon storage in products, and displacement of fossil-intensive alternatives over a 40-year period. For the Housing Scenario, the net climate benefit is 16.8M tCO_2 per year vs. BAU-2. This range of values is close to the range found when compared to Low BAU.

4 REFERENCES

1. D. L. Sanchez, J. H. Nelson, J. Johnston, A. Mileva, D. M. Kammen, Biomass enables the transition to a carbon-negative power system across western North America. *Nat. Clim. Chang.* **5**, 230–234 (2015).

2. M. A. Blasdel, "Decay of woody residues as the counterfactual treatment to mobilization for bioelectricity generation," Humboldt State University. (2020).

3. K. G. Roberts, B. A. Gloy, S. Joseph, N. R. Scott, J. Lehmann, Life cycle assessment of biochar systems: Estimating the energetic, economic, and climate change potential. *Environ. Sci. Technol.* **44**, 827–833 (2010).

4. D. Woolf, J. E. Amonette, F. A. Street-Perrott, J. Lehmann, S. Joseph, Sustainable biochar to mitigate global climate change. *Nat. Commun.* **1**, 1–9 (2010).

5. W. Li, Q. Dang, R. Smith, R. C. Brown, M. M. Wright, Techno-economic analysis of the stabilization of bio-oil fractions for insertion into petroleum refineries. *ACS Sustain. Chem. Eng.* **5**, 1528–1537 (2017).

6. W. Li, Q. Dang, R. C. Brown, D. Laird, M. M. Wright, The impacts of biomass properties on pyrolysis yields, economic and environmental performance of the pyrolysis-bioenergy-biochar platform to carbon negative energy. *Bioresour. Technol.* **241**, 959–968 (2017).

7. J. McKechnie, S. Colombo, J. Chen, W. Mabee, H. L. MacLean, Forest bioenergy or forest carbon? Assessing trade-offs in greenhouse gas mitigation with wood-based fuels. *Environ. Sci. Technol.* **45**, 789–795 (2011).

8. G. Liu, E. D. Larson, R. H. Williams, T. G. Kreutz, X. Guo, Making Fischer-Tropsch fuels and electricity from coal and biomass: Performance and cost analysis. *Energy and Fuels* **25**, 415–437 (2011).

9. X. Xie, M. Wang, J. Han, Assessment of fuel-cycle energy use and greenhouse gas emissions for Fischer-Tropsch diesel from coal and cellulosic biomass. *Environ. Sci. Technol.* **45**, 3047–3053 (2011).

10. M. Puettmann, E. Oneil, Woodlife Environmental Consultants, Cradle to Gate Life Cycle Assessment of Softwood Lumber Production from the Pacific Northwest. 1–35 (2013).

11. C. Smyth, G. Rampley, T. C. Lemprière, O. Schwab, W. A. Kurz, Estimating product and energy substitution benefits in national-scale mitigation analyses for Canada. *GCB Bioenergy* **9**, 1071–1084 (2017).

12. T. Bowers, M. E. Puettmann, I. Ganguly, I. Eastin, Cradle-to-gate life-cycle impact analysis of glued-laminated (glulam) timber: Environmental impacts from glulam produced in the US pacific northwest and southeast. *For. Prod. J.* **67**, 368–380 (2017).

13. C. Antonini, *et al.*, Hydrogen from wood gasification with CCS – a techno-environmental analysis of production and use as transport fuel. *Sustain. Energy Fuels* **5**, 2602–2621 (2021).

14. S. E. Baker, and 20+ Authors, Getting to Neutral: Options for Negative Carbon Emissions in

California. *Lawrence Livermore Natl. Lab. LLNL-TR-796100* (2020).

15. T. B. Jain, J. S. Fried, S. M. Loreno, Simulating the Effectiveness of Improvement Cuts and Commercial Thinning to Enhance Fire Resistance in West Coast Dry Mixed Conifer Forests. *For. Sci* **66**, 157–177 (2020).

16. J. S. Fried, L. D. Potts, S. M. Loreno, G. A. Christensen, R. J. Barbour, Inventory-Based Landscape-Scale Simulation of Management Effectiveness and Economic Feasibility with BioSum. *J. For.* (2016) https:/doi.org/10.5849/jof.15-087.

17. J. S. Fried, S. M. Loreno, B. D. Sharma, C. F. Starrs, W. C. Stewart, "Inventory based landscape-scale simulation to assess effectiveness and feasibility of reducing fire hazards and improving forest sustainability in California with BioSum" (2016).

18. California State Board of Equalization, "Harvest Value Schedule" (2015).

19. BECK Group, CAWBIOM: California Assessment of Wood Business Innovation Opportunities and Markets (2015).

20. R. Mirski, D. Dziurka, The utilization of chips from comminuted wood waste as a substitute for flakes in the oriented strand board core. *For. Prod. J.* **61**, 473–477 (2011).

21. Z. Shareefdeen, A. Elkamel, S. Tse, Review of current technologies used in municipal solid waste-to-energy facilities in Canada. *Clean Technol. Environ. Policy* **17**, 1837–1846 (2015).

22. M. L. Mann, *et al.*, Incorporating anthropogenic influences into fire probability models: Effects of human activity and climate change on fire activity in California. *PLoS One* **11**, 1–21 (2016).

23. C. F. Starrs, V. Butsic, C. Stephens, W. Stewart, The impact of land ownership, firefighting, and reserve status on fire probability in California. *Environ. Res. Lett.* **13** (2018).

24. M. A. Finney, *et al.*, A Method for Ensemble Wildland Fire Simulation. *Environ. Model. Assess.* (2011) https:/doi.org/10.1007/s10666-010-9241-3.

25. J. Campbell, D. Donato, D. Azuma, B. Law, Pyrogenic carbon emission from a large wildfire in Oregon, United States. *J. Geophys. Res. Biogeosciences* **112**, 1–11 (2007).

26. J. E. Stenzel, *et al.*, Fixing a snag in carbon emissions estimates from wildfires. *Glob. Chang. Biol.* **25**, 3985–3994 (2019).

27. B. N. I. Eskelson, V. J. Monleon, J. S. Fried, A 6 year longitudinal study of post-fire woody carbon dynamics in California's forests. *Can. J. For. Res.* **46**, 610–620 (2016).

28. J. L. Campbell, J. B. Fontaine, D. C. Donato, Carbon emissions from decomposition of fire-killed trees following a large wildfire in Oregon, United States. *J. Geophys. Res. Biogeosciences* **121**, 718–730 (2016).

29. K. A. Pietsch, *et al.*, Global relationship of wood and leaf litter decomposability: The role of functional traits within and across plant organs. *Glob. Ecol. Biogeogr.* **23**, 1046–1057 (2014).

30. C. W. Stephens, B. M. Collins, J. Rogan, Land ownership impacts post-wildfire forest regeneration in Sierra Nevada mixed-conifer forests. *For. Ecol. Manage.* **468**, 118161 (2020).

31. S. A. Rebain, The Fire and Fuels Extension to the Forest Vegetation Simulator: Updated Model

Documentation. *United States Dep. Agric. / For. Serv. For. Manag. Serv. Center, Fort Collins, CO*, 403 (2015).

32. B. M. Collins, *et al.*, Modeling hazardous fire potential within a completed fuel treatment network in the northern Sierra Nevada. *For. Ecol. Manage.* (2013) https:/doi.org/10.1016/j.foreco.2013.08.015.

33. B. M. Collins, S. L. Stephens, G. B. Roller, J. J. Battles, Simulating fire and forest dynamics for a landscape fuel treatment project in the Sierra Nevada. *For. Sci.* (2011) https:/doi.org/10.1093/forestscience/57.2.77.

34. J. J. Moghaddas, B. M. Collins, K. Menning, E. E. Y. Moghaddas, S. L. Stephens, Fuel treatment effects on modeled landscape-level fire behavior in the northern Sierra Nevada. *Can. J. For. Res.* (2010) https:/doi.org/10.1139/X10-118.

35. S. L. Stephens, J. J. Moghaddas, Experimental fuel treatment impacts on forest structure, potential fire behavior, and predicted tree mortality in a California mixed conifer forest. *For. Ecol. Manage.* (2005) https:/doi.org/10.1016/j.foreco.2005.03.070.

36. S. L. Stephens, *et al.*, Fire treatment effects on vegetation structure, fuels, and potential fire severity in western U.S. forests. *Ecol. Appl.* (2009) https:/doi.org/10.1890/07-1755.1.

37. N. H. F. French, *et al.*, Model comparisons for estimating carbon emissions from North American wildland fire. *J. Geophys. Res. Biogeosciences* (2011) https:/doi.org/10.1029/2010JG001469.

38. M. A. Finney, Calculation of fire spread rates across random landscapes. *Int. J. Wildl. Fire* (2003) https:/doi.org/10.1071/WF03010.

39. B. M. Collins, J. D. Miller, E. E. Knapp, D. B. Sapsis, A quantitative comparison of forest fires in central and northern California under early (1911-1924) and contemporary (2002-2015) fire suppression. *Int. J. Wildl. Fire* (2019) https:/doi.org/10.1071/WF18137.

40. B. M. Collins, Fire weather and large fire potential in the northern Sierra Nevada. *Agric. For. Meteorol.* **189**–**190**, 30–35 (2014).

41. D. J. Krofcheck, M. D. Hurteau, R. M. Scheller, E. L. Loudermilk, Restoring surface fire stabilizes forest carbon under extreme fire weather in the Sierra Nevada. *Ecosphere* **8** (2017).

42. D. J. Krofcheck, M. D. Hurteau, R. M. Scheller, E. L. Loudermilk, Prioritizing forest fuels treatments based on the probability of high-severity fire restores adaptive capacity in Sierran forests. *Glob. Chang. Biol.* (2018) https:/doi.org/10.1111/gcb.13913.

43. S. Liang, M. D. Hurteau, A. L. R. Westerling, Response of Sierra Nevada forests to projected climate–wildfire interactions. *Glob. Chang. Biol.* **23**, 2016–2030 (2017).

44. S. Liang, M. D. Hurteau, A. L. Westerling, Large-scale restoration increases carbon stability under projected climate and wildfire regimes. *Front. Ecol. Environ.* **16**, 207–212 (2018).

45. G. A. Christensen, A. N. Gray, O. Kuegler, N. A. Tase, M. Rosenberg, “AB 1504 California Forest Ecosystem and Harvested Wood Product Carbon Inventory: 2017 Reporting Period. Final Report.” (2019).

46. D. B. McKeever, J. L. Howard, Solid Wood Timber Products Consumption in Major End Uses in the

United States, 1950–2009 (2010).

47. A. Geng, J. Chen, H. Yang, Assessing the Greenhouse Gas Mitigation Potential of Harvested Wood Products Substitution in China. *Environ. Sci. Technol.* **53**, 1732–1740 (2019).

48. K. E. Skog, Sequestration of carbon in harvested wood products for the United States. *For. Prod. J.* **58**, 56–72 (2008).

49. W. C. Stewart, G. M. Nakamura, Documenting the full climate benefits of harvested wood products in northern california: Linking harvests to the us greenhouse gas inventory. *For. Prod. J.* **62**, 340–353 (2012).

50. X. Wang, J. M. Padgett, F. B. De La Cruz, M. A. Barlaz, Wood biodegradation in laboratory-scale landfills. *Environ. Sci. Technol.* **45**, 6864–6871 (2011).

51. E. Baik, *et al.*, Geospatial analysis of near-term potential for carbon-negative bioenergy in the United States. *Proc. Natl. Acad. Sci. U. S. A.* **115**, 3290–3295 (2018).

52. US EPA, eGRID Summary Tables 2016. *eGRID* (2018).

53. Gas Technology Institute, "Low-Carbon Renewable Natural Gas (RNG) from Wood Wastes" (2019).

54. E. D. Larson, H. Jin, F. E. Celik, Large-scale gasification-based coproduction of fuels and electricity from switchgrass. *Biofuels, Bioprod. Biorefining* (2009) https:/doi.org/10.1002/bbb.137.

55. P. A. Meyer, L. J. Snowden-Swan, S. B. Jones, K. G. Rappé, D. S. Hartley, The effect of feedstock composition on fast pyrolysis and upgrading to transportation fuels: Techno-economic analysis and greenhouse gas life cycle analysis. *Fuel* **259**, 116218 (2020).

56. W. F. Lazarus, D. G. Tiffany, R. S. Zalesny, D. E. Riemenschneider, Economic impacts of short-rotation woody crops for energy or oriented strand board: A Minnesota case study. *J. For.* **109**, 149–156 (2011).
